# Drug Repurposing of Metformin for the Treatment of Haloperidol-Related Behavior Disorders and Oxidative Stress: A Preliminary Study

**DOI:** 10.3390/pharmaceutics16030403

**Published:** 2024-03-15

**Authors:** George Jîtcă, Zsolt Gáll, Carmen-Maria Jîtcă, Mădălina-Georgiana Buț, Erzsébet Májai

**Affiliations:** 1 Department of Pharmacology and Clinical Pharmacy, Faculty of Pharmacy, George Emil Palade University of Medicine, Pharmacy, Science and Technology of Târgu Mureș, 540139 Târgu Mureș, Romania; george.jitca@umfst.ro; 2 Doctoral School of Medicine and Pharmacy, Institution Organizing Doctor’s Degree University Studies, George Emil Palade University of Medicine, Pharmacy, Science and Technology of Târgu Mureș, 540139 Târgu Mureș, Romania; carmenrusz20@gmail.com; 3 Department of Biochemistry, Faculty of Pharmacy, George Emil Palade University of Medicine, Pharmacy, Science and Technology of Târgu Mureș, 540139 Târgu Mureș, Romania; madalina-georgiana.batrinu@umfst.ro; 4 Department of Toxicology and Biopharmacy, Faculty of Pharmacy, George Emil Palade University of Medicine, Pharmacy, Science and Technology of Târgu Mureș, 540139 Târgu Mureș, Romania; erzsebet.fogarasi@umfst.ro

**Keywords:** oxidative stress, cognition, metformin, malondialdehyde, glutathione

## Abstract

A particular attribute of the brain lies in the ability to learn, acquire information from the environment, and utilize the learned information. Previous research has noted that various factors (e.g., age, stress, anxiety, pathological issues), including antipsychotic medications, affect the brain and memory. The current study aimed to reveal the effects of chronic metformin treatment on the cognitive performance of rats and on commonly measured markers for oxidative stress. Wistar male rats (n = 40) were randomly divided into four groups: CTR (n = 10)–control group, METF (n = 10)–animals receiving metformin 500 mg/kg, HAL (n = 10)–animals receiving haloperidol 2 mg/kg, and HALMETF (n = 10)–animals receiving haloperidol 2 mg/kg and metformin 500 mg/kg. The medication was administered daily by oral gavage for 40 days. Memory and learning were assessed using the Morris Water Maze (MWM) test. At the end of the MWM, the rodents were decapitated under anesthesia, and the brain and blood samples were assayed by liquid chromatography for markers of oxidative stress (malondialdehyde, MDA, reduced/oxidized glutathione ratio, GSH/GSSG). The quantification of brain-derived neurotrophic factor (BDNF) was performed using the conventional sandwich ELISA technique. In the HALMETF group, metformin attenuated the negative effects of haloperidol. Brain and plasma MDA levels increased in the HAL group. Brain and plasma GSH/GSSG ratios and BDNF levels did not reveal any differences between groups. In conclusion, metformin treatment limits the deleterious cognitive effects of haloperidol. The effect on oxidative stress markers may also point toward an antioxidant-like effect of metformin, but this needs further tests for confirmation.

## 1. Introduction

Numerous preclinical studies suggest the use of metformin to ameliorate the symptoms of neurodegenerative diseases due to its neuroprotective effect [1,2,3,4,5,6]. In addition, the idea of using it in other age-related diseases has been suggested [7]. Regarding the mechanism of action of metformin, there is much controversy, but the activation of AMPK is the result of the interaction of the biguanide compound with the mitochondrial complex I [8,9,10]. However, metformin is also associated with the modulation of other cellular signaling pathways, including caspase 3, Bcl-2, and mTOR pathways, which may explain the anti-apoptotic, antioxidant, and pro-autophagic effects [11]. There are data that highlight its aforementioned effects on the brain due to its property of crossing the blood–brain barrier [12,13], thus promoting neurogenesis and neurotrophin synthesis [14], improving spatial memory [15,16], having antioxidant effects [17], and being neuroprotective with inhibition of neuronal apoptosis [18], with positive results in the case of models of neurodegenerative diseases [19].

Memory and learning are essential functions for good psychosocial integration, allowing one to recognize past experiences to improve future choices and actions, while previous research has demonstrated dopamine’s involvement in memory formation [20]. Also, previous research has noted that various factors (age, stress, anxiety, pathological issues), including antipsychotic medications, affect the brain and memory [21,22,23,24,25,26,27,28].

For mental health disorders (delusions and hallucinations) and schizophrenia, antipsychotics were developed as a therapeutic class. Thus, for positive symptomatology, haloperidol is one of the most prescribed therapeutic alternatives [29], although it is a first-generation neuroleptic and has well-known extrapyramidal side effects [30], and blocking D_2_ receptors also impacts working memory [31]. However, long-term treatment is associated with various unwanted effects, both from the motor sphere (tardive dyskinesia, akathisia, bradykinesia) and from the psychosocial sphere, leading to the impairment of memory and cognitive abilities [32].

Therefore, it is hypothesized that dopamine has cortical and hippocampal implications [33], and the inhibition of dopaminergic neural circuits directly influences memory, the ability to learn, and the ability to use accumulated information [34] via D_1_ and D_2_ dopaminergic receptors [35]. Furthermore, studies on experimental animals demonstrated the decline of cognitive performance after the intrahippocampal injection of D_2_ antagonists, followed by the attenuation of these performances as a result of the administration of D_2_ agonists, supporting the hypothesis of the involvement of dopamine in the previously mentioned processes (memory, learning) [36].

Due to the increased turnover of dopamine, the level of oxidative stress in the brain is increased, and this effect is mainly observed in the hippocampal neurons [37,38,39]. In addition, classical neuroleptics, including haloperidol, are known to generate reactive species, with multiple negative neurological effects. Given the above, the cognitive and memory deficits that occur in the treatment of classical antipsychotics should be taken into account, as well as the influence of oxidative stress on these evolutionary features [40].

Essential roles in the pathophysiology of these phenomena related to oxidative stress are the accumulation of molecules resulting from oxidative processes (lipid peroxidation), such as malondialdehyde (MDA)**,** and the alteration of the reduced glutathione/oxidized glutathione ratio (GSH/GSSG). Multiple studies claim that antipsychotics, particularly haloperidol, cause adverse effects due to oxidative stress. Thus, an attempt was made to alleviate oxidative stress by administering betaine, rice bran oil, or cannabidiol [41,42,43]. These studies confirm that the long-term use of haloperidol disrupts the balance of antioxidant systems, while the above-mentioned compounds alleviate the adverse effects and oxidative stress generated.

A decrease in brain-derived neurotrophic factor expression was observed with haloperidol use [44,45]. BDNF has been shown to play an important role in neurogenesis, neuronal plasticity, and memory formation [46,47].

The main aim of the study was to evaluate the effects of metformin administration in chronic haloperidol treatment, on memory and its possible antioxidant effects by determining the level of MDA and GSH/GSSG, and to evaluate whether metformin could be an option for the management of cognitive decline and oxidative stress in chronic haloperidol treatment.

## 2. Materials and Methods

### 2.1. Animals and Treatment

In order to test the hypothesis, 40 male Wistar rats (430–450 g) from our university’s animal facility were allocated randomly into four distinct groups: CTR (n = 10), which served as control group and received distilled water, METF (n = 10), which recieved metformin at a dosage of 500 mg/kg, HAL (n = 10), which received haloperidol at a dosage of 2 mg/kg, and HALMETF (n = 10), which received a combination of haloperidol at 2 mg/kg and metformin at 500 mg/kg. Animals were randomized with the aid of a computer-based random order generator. Treatments were administrated orally via gavage, adjusting the dosage according to each animal’s pre-measured body weight. The regimen continued for 40 consecutive days, with each dose administered in a volume of 1mL/kg, conducted in a designated separate room. Dose selection for haloperidol was based on previous reports that demonstrated the occurrence of oxidative stress by affecting several antioxidant enzymes in the brain [48]. For metformin, previous studies have also confirmed the antioxidant effects in rats at a dose of 500 mg/kg [49,50,51,52].

The animals were acclimatized for a period of 7 days by being handled daily for stress reduction before the start of the experiment. The environmental conditions were standard (12/12 h light–dark cycle, temperature 20 ± 2 °C, 60% ± 10% humidity) with food and water ad libitum. For dosing adjustments, body weight was recorded once a week. All experimental procedures complied with European Directive 2010/63/EU and were approved by the Ethics Committee for Scientific Research of the George Emil Palade University of Medicine, Pharmacy, Science and Technology of Targu Mures (approval no. 533/22.11.2019) and by National Sanitary Veterinary and Food Safety Authority (approval no. 42/2020). All measures have been taken to minimize the suffering of the animals.

**Figure 1:**
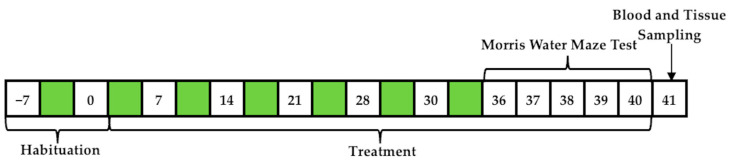
Schematic representation of the chronology of the experimental design.

This study was designed to assess and correlate the long-term effects of haloperidol use with metformin on cognition, memory, and learning. The timeline in Figure 1 shows the tests performed for testing memory, learning, and coordination skills in order to determine cognitive abilities.

### 2.2. Chemicals and Reagents

Haloperidol (Haloperidol Richter 2 mg/mL, Gedeon Richter, Targu Mures, Romania) and metformin (Glucophage, 500 mg/tablet, Merck Santé, Semoy, France) were acquired from the domestic pharmaceutical market. Haloperidol oral solution contained methyl p-hydroxybenzoate, n-propyl p-hydroxybenzoate, and lactic acid as inactive ingredients. The metformin tablets were composed of carmellose sodium, hypromellose, and magnesium stearate. Powdered tablets were added to distilled water that served as a solvent, resulting in an extempore suspension. Metformin is highly soluble in water (>300 mg/mL) at room temperature. As a result, the extempore suspension from tablets was homogeneous in terms of metformin content. For the HPLC analysis, the same reagents as those used for the validation of the determination methods were used.

### 2.3. Behavioral Assessment

The behavioral assessment was performed 8 h after the medication was administered in order to eliminate the sedative and cataleptic effects of haloperidol. The investigators were not blinded to animal groups during experiments, but in order to reduce bias, the offline behavioral analysis was performed by other two experienced researchers to assess inter-observer reliability.

#### Morris Water Maze

This method of assessment was chosen because it requires an intact hippocampal function, involving several important processes for memorization and learning (coding of information, consolidation, preservation, and reuse). The maze consisted of a circular pool (60 cm in height, 90 cm in diameter) filled with water to a depth of 35 cm and maintained at room temperature (25 ± 1) °C. The pool was divided into four equal quadrants with printed geometric cues placed in the test chamber as clues. On the first day, a platform with a diameter of 9 cm was submerged 1 cm below the water level in the middle of a specific quadrant, and the rats were subjected to four trials per day for four consecutive days (roughly the same time each day), being forced to find the platform in 120 s with an interval between trials of 60 s [53]. If the rat managed to find the platform in 120 s, it was allowed to stay on it for 30 s. Animals that failed to find the platform were guided to it and allowed to remain there for 30 s. The starting position was changed for each trial, and quadrant 4 (Q4) was considered to be the place of the fixed platform [54]. The experimental design is shown in Table 1. On the fifth day, the probe test was performed, in which the platform was removed and each rat was placed for 120 s from the opposite quadrant where the platform was positioned. Regarding the time spent in the target quadrant and the number of crossings through the target quadrant, the 120 s were used in the statistical analysis (distance traveled, swimming speed, frequency of entries into the target quadrant, etc.). During the 4 days of training, the reference memory was tested, and on the last day, the spatial memory and retrieval capabilities were tested. The activity of each rat was monitored using a top-view camera at 30 fps. All trials were analyzed with EthoVision XT (Noldus IT, Wageningen, The Netherlands, version 11.5).

### 2.4. Experimental Procedures for Collecting Samples

After the MWM test, the rodents were decapited under anesthesia with ketamine/xylazine in a dose mixture of ketamine (100 mg/kg) and xylazine (10 mg/kg). Their brains were rapidly excised, washed with ice-cold saline solution (0.9% NaCl), weighed, immersed in liquid nitrogen, and stored at −80 °C for further analysis. Trunk blood samples were collected upon decapitation in K3 EDTA-coated tubes and centrifuged using a cooled centrifuge (4 °C) at 3500 rpm for 15 min. The obtained plasma was transferred to polyethylene tubes and maintained at −80 °C until analysis.

#### 2.4.1. Determination of MDA

The degree of lipid peroxidation was determined by measuring brain and plasma MDA levels. MDA was determined by measuring thiobarbituric reactive species, combined with an HPLC method, according to the method that was previously reported by our group [55].

Briefly, for MDA analysis, brains were homogenized for 5 min in IKA Ultra-Turrax Tube Drive (Königswinter, Germany). After homogenization, for protein precipitation, acetonitrile (ACN) was added (1:3, *v*/*v*). The samples underwent centrifugation at 10,000× *g* for 10 min, and the resulting supernatant was then diluted with pure water at 1:1 ratio, *v*/*v*. Subsequently, 600 μL thiobarbituric acid in concentration of 4 mg/mL and 1000 μL solution of sulfuric acid in concentration of 2.66 μL/mL were added to 400 μL sample. The mixture was heated at 100 °C for 60 min using a TS-100C, Thermo-Shaker (BioSan, Riga, Latvia). Following the heating process, the samples were transferred in HPLC vials and promptly analyzed following the derivatization reaction. For the analysis of plasma samples, a similar procedure was followed, with strict modifications [56]. Representative chromatograms of plasma and brain MDA analysis are shown in Supplementary Figures S1 and S2.

#### 2.4.2. Determination of GSH/GSSG Ratio

The quantification of GSH/GSSG ratio was carried out according to a validated method previously reported by our group [57]. Brains were homogenized (for 5 min) in the previously mentioned apparatus and centrifuged (10,000× *g* for 10 min). The samples underwent centrifugation, and following this step, 500 μL supernatant was retrieved and mixed with 500 μL Ellman’s reagent. For GSSG samples, the mixture was heated for 60 min at 80 °C in the same device as previously mentioned. Meanwhile, GSH samples were left at room temperature for 10 min. Subsequently, 300 μL of TCA 20% was added to both the GSH and GSSG sample series. The samples were centrifuged at 13,000× *g* for 10 min. Following centrifugation, the supernatant was carefully retrieved and transferred into HPLC vials. Representative chromatograms of plasma and brain MDA analysis are shown in Supplementary Figures S3 and S4.

#### 2.4.3. Immunoassay for BDNF

Quantification of brain-derived neurotrophic factor (BDNF) was performed using conventional sandwich ELISA technique (ELISA Dynex DSX), according to the manufacturer’s instructions (0266F0750, Sigma-Aldrich, St. Louis, MO, USA). The concentration of samples was extrapolated from a standard curve (R^2^ = 0.98).

### 2.5. Statistical Analysis

The obtained data were evaluated with GraphPad Prism software (San Diego, CA, USA, ver. 8). Shapiro–Wilk test was used to assess the normality of data. One-way analysis of variance (ANOVA) followed by Tukey’s multiple comparison post-hoc test was used for analyzing continuous parameters that were normally distributed. Two-way ANOVA was applied for repeated-measure parameters. Kruskal–Wallis test followed by Dunn’s multiple comparison test was performed over the one-way ANOVA test where the raw data was not normally distributed. Normally distributed data was expressed as mean ± SEM; otherwise, median with interquartile range was used. The significance level was set at *p* < 0.05.

## 3. Results

All animals survived the 40-day exposure to haloperidol and metformin and were sacrificed according to the schedule. The clinical signs of the animals did not show behavioral alterations. Throughout the study, body weight and blood glucose levels were monitored, but no changes were observed. Body weight changes were included in Supplementary Table S1.

### 3.1. Morris Water Maze

#### 3.1.1. Escape Latency

**Figure 2:**
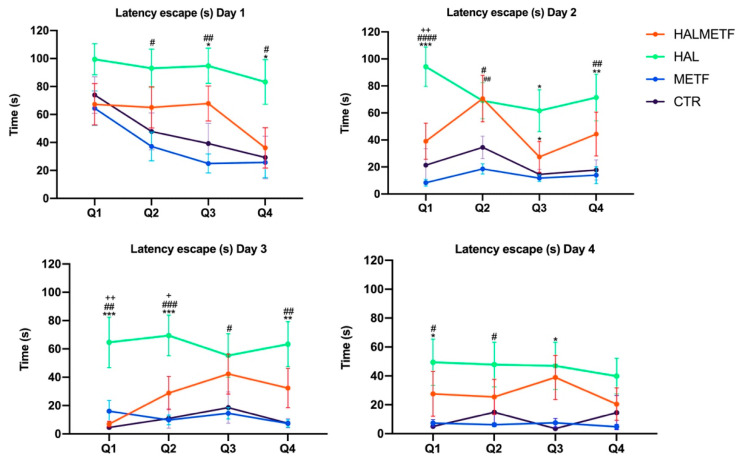
The escape latency through the four days of practice. Values displayed are means ± SEM. Statistically significant differences compared to the CTR group (n = 10) are noted with * *p* < 0.05, ** *p* < 0.01, *** *p* < 0.001. Statistically significant differences compared to the METF group (n = 10) are noted with ^#^ *p* < 0.05, ^##^ *p* < 0.01, ^###^ *p* < 0.001, ^####^ *p* < 0.0001. Statistically significant differences compared to the HALMETF group (n = 10) are noted with ^+^ *p* < 0.05, ^++^ *p* < 0.01.

The results of the escape latency are illustrated in Figure 2. On day 1, both the treatment effect F(3, 144) = 13.31, *p* < 0.0001 and the quadrant effect F(3, 144) = 4.258, *p* = 0.0065 were significant. On days 2, 3, and 4, only the treatment effect was significant: F(3, 144) = 22.1, *p* < 0.0001; F(3, 144) = 21.22, *p* < 0.0001, and F(3, 144) = 11.27, *p* < 0.0001, respectively. Following the post-hoc tests, it was revealed that the HAL and HALMETF groups initially exhibited higher average latency. However, this latency decreased, with HALMETF group demonstrating significantly better performance compared to HAL (day 2, Q1: 94.26 ± 14.56 vs. 39.07 ± 13.33, *p* = 0.0054; day 3, Q1: 64.61 ± 17.74 vs. 6.96 ± 1.9, *p* = 0.001; day 3, Q2: 69.49 ± 14.31 vs. 28.88 ± 11.69, *p* = 0.0387.

#### 3.1.2. Distance in Quadrant

**Figure 3:**
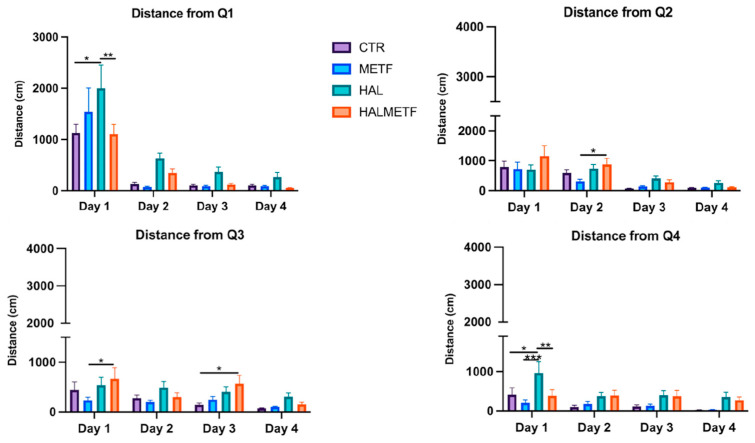
The distances covered to the rescue platform in the four quadrants, during the four days of training. Values displayed are means ± SEM. Statistically significant differences are noted with * *p* < 0.05, ** *p* < 0.01, and *** *p* < 0.001.

The distances traveled by the animals can be visualized in Figure 3. It can be seen that the longer distances were traveled by the rats in the HAL and HALMETF groups. Differences in statistical importance can be observed, so the following results can be presented: quadrant 1, treatment effect F(3, 134) = 4.914, *p* = 0.0029; day effect F(3, 134) = 42.11, *p* < 0.0001; quadrant 2, treatment effect F(3, 137) = 3.211, *p* = 0.0250; day effect F(3, 137) = 20.11, *p* < 0.0001; quadrant 3, treatment effect F(3, 135) = 5.986, *p* = 0.0007; day effect F(3, 135) = 6.086, *p* = 0.0006; quadrant 4, treatment effect F(3, 134) = 8.258, *p* < 0.0001; day effect F(3, 134) = 4.634, *p* = 0.0041. It is observed that the differences tend to decrease for each treated group, regardless of the starting quadrant or the training day.

#### 3.1.3. Swim Speed

**Figure 4:**
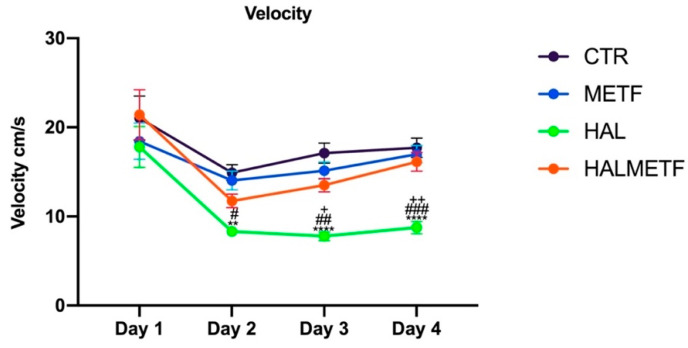
The effect of HAL and METF treatment on swimming speeds during the four days of training. Values displayed are means ± SEM. Statistically significant differences compared to the CTR group are noted with ** *p* < 0.01, **** *p* < 0.0001. Statistically significant differences compared to the METF group are noted with ^#^ *p* < 0.05, ^##^ *p* < 0.01, ^###^ *p* < 0.0001. Statistically significant differences compared to the HALMETF group are noted with ^+^ *p* < 0.05, ^++^ *p* < 0.01.

The swimming speed was also analyzed in the four days of training by averaging the speeds after release from each quadrant, as can be seen in Figure 4. Differences in statistical importance can be observed based on statistical determinations: treatment effect F(3, 623) = 18.03, *p* < 0.0001, day effect F(3, 623) = 20.76, *p* < 0.0001. Except for the first day of training, where there were also the highest speeds of swimming, probably due to the stress of the new environment and the ignorance of the task, statistically significant differences can be observed. The HAL group had lower speeds than the HALMETF group on both day 3 (*p* < 0.05) and day 4 (*p* < 0.01).

#### 3.1.4. Probe Trial

**Figure 5:**
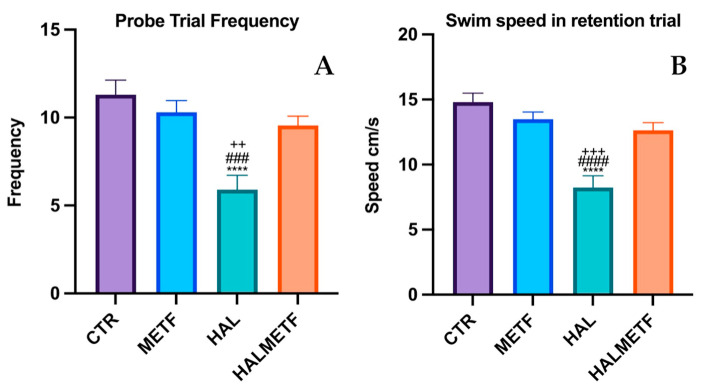
Differences in entries frequency in Q4 (**A**) and swimming speed (**B**). Values displayed are means ± SEM. Statistically significant differences compared to the CTR group are noted with **** *p* < 0.0001. Statistically significant differences compared to the METF group are noted with ^###^ *p* < 0.001, ^####^ *p* < 0.0001. Statistically significant differences compared to the HALMETF group are noted with ^++^ *p* < 0.01, ^+++^ *p* < 0.001.

**Figure 6:**
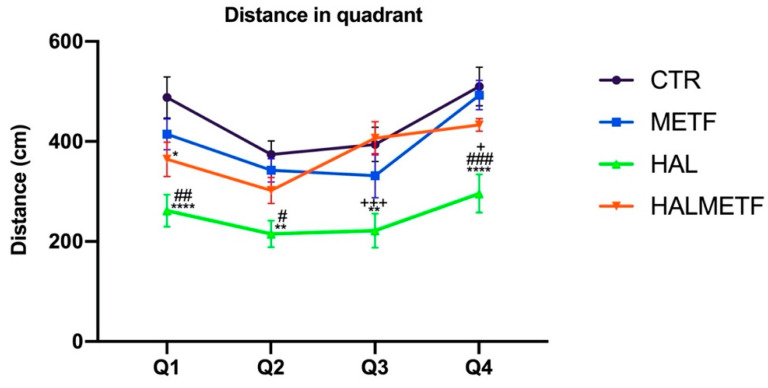
The distances traveled by the four groups in each quadrant. Values displayed are means ± SEM. Statistically significant differences compared to the CTR group are noted with * *p* < 0.05, ** *p* < 0.01, **** *p* < 0.0001. Statistically significant differences compared to the METF group are noted with ^#^ *p* < 0.05, ^##^ *p* < 0.01, ^###^ *p* < 0.001. Statistically significant differences compared to the HALMETF group are noted with ^+^ *p* < 0.05, ^+++^ *p* < 0.001.

**Figure 7:**
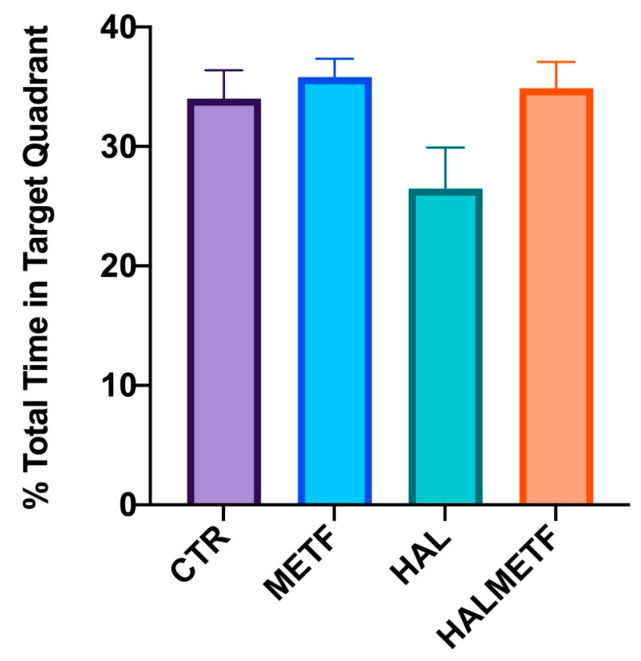
The effect of chronic treatment on time spent in the target quadrant, Q4. Values displayed are means ± SEM.

On the probe day, statistically significant differences (*p* < 0.05) were observed between the groups of interest in terms of number of crossings through the target quadrant (Figure 5A). Two things were noted: one is that all groups showed significant differences compared to the HAL group, which had the lowest frequency of crossing through the target quadrant, and the second observation was that the HALMETF group showed no differences compared to CTR and METF groups. A similar trend was also observed for swimming speed, as shown in Figure 5B. Another parameter followed was the distance traveled in the target quadrant, in which case, the same trend as the ones for the parameters discussed above was found (Figure 6), F(3, 144) = 25.95, *p* < 0.0001; quadrant F(3, 144) = 11.17, *p* < 0.0001. All four groups (CTR, METF, HAL, HALMETF) spent approximately the same time in the target quadrant, with the time being lower in the case of the HAL group but without statistical differences, as can be seen in Figure 7.

### 3.2. Biochemical Parameters

#### 3.2.1. Plasma and Brain Level of MDA

**Figure 8:**
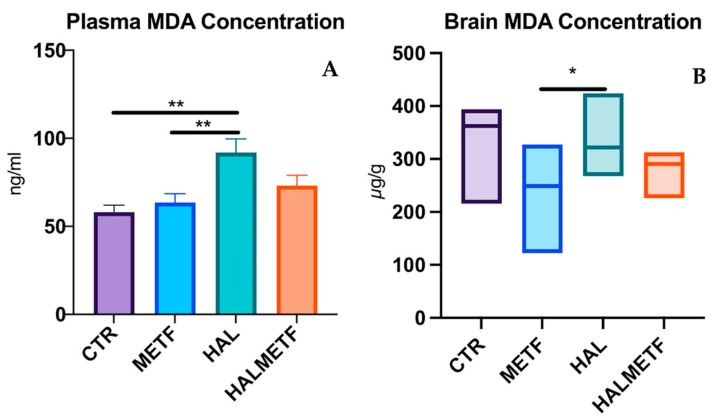
Effects of haloperidol and metformin on plasma (**A**) and brain (**B**) malondialdehyde (MDA) levels. Values displayed are means ± SEM for plasma MDA concentration and median (IQR) for brain MDA concentrations; * *p* < 0.05, ** *p* < 0.01.

MDA is the most common marker of oxidative stress, and the impact of haloperidol and metformin administration on plasma and brain MDA levels was analyzed. Significant differences were noted between the plasma levels of MDA among the experimental groups of rats, *p* < 0.01, as shown in Figure 8A. In addition, significant differences were also observed between MDA levels in the brain, *p* < 0.05.

#### 3.2.2. Plasma and Brain GSH/GSSG Ratio

**Figure 9:**
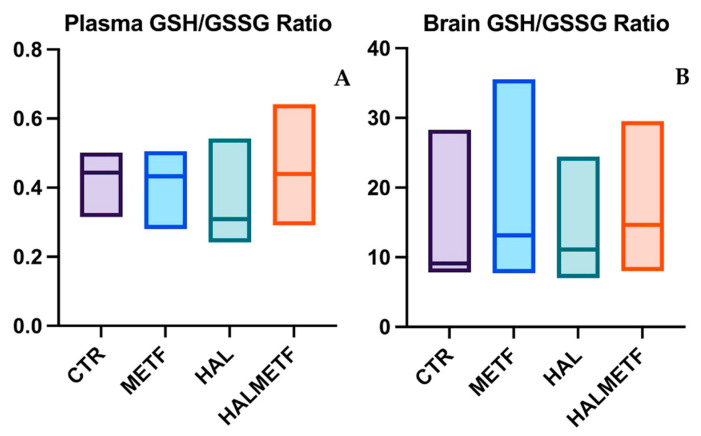
Effects of haloperidol and metformin on plasma (**A**) and brain (**B**) GSH/GSSG ratios. Values displayed are median (IQR).

No significant differences were observed among the experimental groups regarding the GSH/GSSG ratio, both in the plasma and brain tissue homogenate. The results indicated that glutathione metabolism remained unchanged, regardless of the applied treatment, as shown in Figure 9.

#### 3.2.3. Plasma Level of BDNF

**Figure 10:**
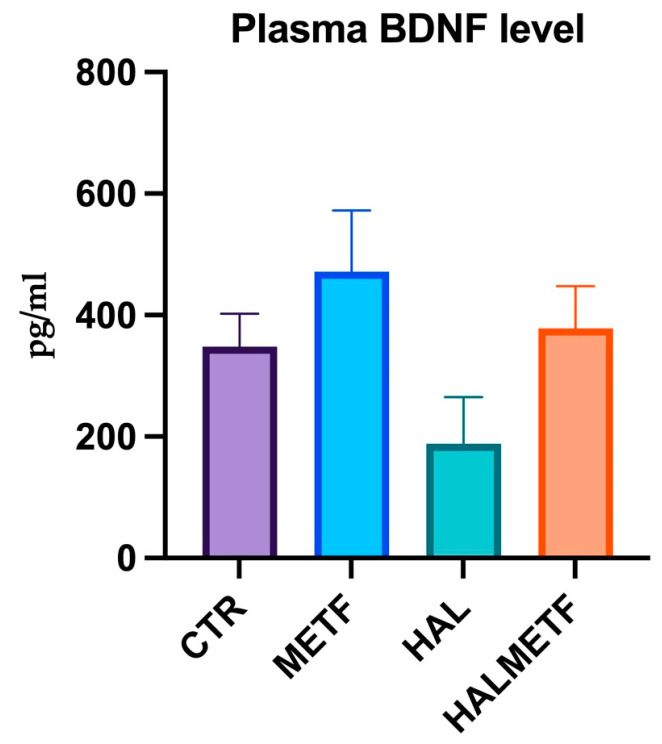
Plasmatic levels of Brain Derived Neurotrophic Factor (BDNF) among the experimental groups of rats. Values displayed are means ± SEM.

Following the determinations made using the ELISA technique, no significant differences were observed. However, lower BDNF levels were found in the haloperidol-treated group compared to the other groups (Figure 10).

## 4. Discussion

The present experimental work was designed to investigate the potential antioxidant effect of metformin on haloperidol-induced oxidative stress and memory deficits in rats. Metformin acts by blocking the mitochondrial complex I [8] and decreasing the production of ROS [58]. We have found that metformin was able to attenuate haloperidol-induced oxidative stress. This effect was observed on the antioxidant parameters measured, i.e., plasma and brain MDA levels. On the other hand, the MWM test showed that metformin improved learning and memory in rats, which is consistent with previous studies [59,60]. Haloperidol treatment was demonstrated to increase MDA levels, decrease antioxidant enzyme activity such as GPx, and alter the GSH/GSSG ratio, as reported earlier [61,62]. In the present study, a significant increase in plasma and brain MDA was found in rats receiving haloperidol treatment. However, the GSH/GSSG ratio showed no differences between groups. The mechanism of action of haloperidol is based on the continuous blockade of D_2_ receptors, to which the blockade of adrenergic, cholinergic, and histaminergic neurotransmissions is added [63]. Therefore, the administration of haloperidol accelerates oxidative processes, leading to oxidative stress. This was also observed in other studies where rats received haloperidol even for shorter periods (21 and 31 days) [64,65]. In a study by Dhingra et al., 21-day treatment decreased glutathione levels [66]. Conversely, in a study conducted by Pillai et al., rats chronically treated with haloperidol showed no changes in GPx activity, as observed in the current study [67], while in other studies, chronic treatment decreased GPx activity [61,68]. Overall, supplementation with antioxidants targeting glutathione metabolism could be a potential therapeutic intervention, as an alteration of the balance of this compound maintains the oxidative state. Multiple studies have shown the anti-inflammatory and neuroprotective effects of metformin [69,70], with one of these mechanisms being based on the ability to limit the production of ROS and/or to scavenge free radicals [58,71]. At the same time, through the antioxidant effect, memory deficits could be prevented, a fact also demonstrated in a study in which metformin promoted the proliferation and survival of neurons associated with memory improvement [72].

It has been established that lipid peroxidation is indicative of oxidative stress due to increased brain lipid content, but the status of GSH homeostasis, which is crucial to cellular defense, remains unclear. The GSH/GSSG ratio, a key antioxidant measure, was not significantly altered in whole brain tissue homogenate in the current study, possibly due to regional variations. Contrasting findings in studies analyzing specific brain regions like the hippocampus or striatum underscore the importance of localized assessments [73,74,75,76,77]. Also, if, in the case of human samples, the level of GSH in the brain can vary pre- and post-mortem, the same can happen in experimental animals [78].

Measuring the GSH/GSSG ratio in blood primarily reflects extracellular (plasma) sources synthesized by the liver, hence serving as a gauge for hepatic antioxidant capacity [79,80]. In erythrocytes, intracellular GSH synthesis occurs, offering heightened sensitivity to disruptions in GSH homeostasis, making intracellular GSH measurement more accurate. Notably, GSH exhibits hormetic responses, potentially enhancing activity in the presence of oxidative stressors like haloperidol. This response involves the activation of pathways such as the pentose phosphate pathway, ensuring GSH levels necessary for antioxidant enzyme function [81,82,83,84]. Similar ratios despite haloperidol administration suggest a preserved physiological response to maintain redox balance. Thus, while the GSH/GSSG ratio is indicative of oxidative stress, its assessment during stress exposure remains challenging due to intricate regulatory mechanisms. However, complexities in assessing GSH levels during oxidative stress warrant further investigation.

The MWM test relies on spatial and recognition memory, which are associated with hippocampus and prefrontal cortex functions. In the current study, behavioral analysis showed that haloperidol-treated animals had lower performances in spatial and recognition memory, demonstrated through longer distances traveled to the escape platform, increased mean latency to escape, and decreased number of platform area crossings. Consistent with the present study, previous research has shown that haloperidol treatment negatively affects memory in rodents [85,86,87]. However, for the rats from the HALMETF group, metformin attenuated the negative effects of haloperidol, demonstrated by the rats’ ability to learn the task. Interestingly, this group exhibited a shorter escape latency, indicating an improvement in spatial learning. The mean number of crossings and the percentage of time spent in the target quadrant were both enhanced. Taken together, metformin treatment could enhance memory impairments induced by haloperidol. As previously reported, haloperidol-induced memory impairment is associated with an impairment of neurogenesis [88]. BDNF was shown to be a crucial factor for neurogenesis [89,90]. Also, another study suggested that loss of BDNF function in the hippocampus is associated with impairment of spatial learning [91,92]. In the present paper, a slight reduction of BDNF in haloperidol-treated rats was noted, but no significant difference was observed between groups.

Understanding the role of BDNF in neuroplasticity has led to various strategies to enhance its expression, such as BDNF infusions, exercise, enriched environments, and metformin [93,94,95,96]. However, the effectiveness of these approaches appears to be influenced by stressors like social isolation, with the type and duration of stress playing a crucial role [97,98]. Peripheral measurements of BDNF may not accurately reflect brain levels due to variations in detecting pro and mature forms [99]. The relationship between BDNF expression and oxidative stress is complex and inconsistent across studies. The histone H3 acetylation of the BDNF gene could shed light on regulatory mechanisms, given the importance of histone modifications in gene expression [100]. Despite efforts, some studies yield inconclusive results on BDNF expression, underscoring the need for further research [101,102,103,104,105]. The results of our study regarding the lack of significant differences in BDNF are in line with those obtained in another study [106], with the difference that in the present work, cognitive changes were observed. Also, similar results, without significant differences in BDNF expression, were obtained in a six-week study [107]. Nevertheless, while progress has been made in understanding BDNF modulation, comprehensive investigations are necessary to fully unravel its complexities and explore its therapeutic potential.

Thus, regardless of the cell type, AMPK activation occurs by inhibiting the mitochondrial complex I. By means of AMPK, peroxisome proliferator-activated receptor λ coactivator (PGC1-α) is phosphorylated and deacetylated with the help of sirtuin1 (SIRT1), thus increasing the activity of PGC-1α, which activates nuclear factor erythroid 2-related factor (Nrf2). Nrf2 translocates into the nucleus, where it attaches to the antioxidant-responsive element (ARE), increasing endogenous antioxidant activity [108]. Associated with this mechanism is the blocking of nuclear factor-kappa B (NF-kB), preventing the synthesis of pro-inflammatory factors that generate oxidative stress [4]. The mammalian target of the rapamycin (mTOR) pathway is involved in the pathogenesis of neurodegenerative diseases and schizophrenia, without being limited to them [109]. Also, on the AMPK pathway, metformin inhibits mTOR complex 1 (mTORC1) and favors autophagy (increases the concentration of beclin-1, the protein important in the formation and maturation of the autophagosome ATG5, the protein involved in the formation of autophagy vesicles) [110,111]. Autophagy is important for memory formation in hippocampus neurons [112,113]. Therefore, the mitigation of the negative effects of haloperidol by metformin is due to a combination of antioxidant, anti-inflammatory factors, mTOR inhibitors, and autophagy promoters, with AMPK at the center of them. However, it is out of the scope of the current study to discuss the potential benefits of associating metformin with haloperidol treatment in patients.

## 5. Limitations of the Study

Although the study design focused on identifying optimal experimental conditions, aspects that limited the study were also identified. Despite efforts to minimize stress during drug administration via gavage, it remains a source of physical discomfort and psychological stress for animals. For this reason, the study had a period of seven days in which the animals were acclimated to handling and gavage. Throughout the study, the well-being of the animals and their state of health were monitored. Individual housing induced social isolation stress, potentially influencing results affecting behavior responses. Socially isolated animals exhibit reduced exploration opportunities. Thus, this can impact the BDNF level. On the other hand, constraints on the number of animals used limited statistical representation within groups. Including a larger number of subjects would enhance representativeness, support statistical power, and diminish random errors, rendering the study more robust. Additionally, a larger sample size reduces individual variability, facilitating clearer data observation. In addition, in the present study, AMPK levels were not determined. By including the analysis of AMPK expression, a correlation between its activation by metformin and the influence on oxidative stress markers can be achieved. Regarding the analysis of markers of oxidative stress, the analysis being carried out on the whole brain reduces the possibility of observing the existence of some differences between treatments, due to functional variation. Thus, a clearer picture of the impact of oxidative stress and how different functions are influenced can be obtained. Further studies are needed to confirm the antioxidant effect of metformin, whether there are other markers influenced, such as mitochondrial permeability, and the enzyme activity of the most important antioxidant enzymes (SOD, CAT, GPx, GSHr). In addition to oxidative stress, caspase activity should be evaluated in order to assay if any apoptosis process occurs. Also, a histological study should be included in order to identify whether there are modifications in the normal morphology of specific brain regions.

## 6. Conclusions

In summary, the present study showed that haloperidol treatment is linked to oxidative stress, demonstrated through significant lipid peroxidation in chronic treatment. However, metformin decreased plasma and brain MDA concentrations and improved some memory function. Further studies on the age, dose, and duration of metformin use are needed to clearly uncover the effects of chronic metformin treatment on haloperidol-induced cognitive deficits. 

## Figures and Tables

**Table 1:** Pool placement sequence by day, during the training period.

Day 1	Q1	Q2	Q3	Q4
Day 2	Q2	Q3	Q4	Q1
Day 3	Q3	Q4	Q1	Q2
Day 4	Q4	Q1	Q2	Q3

## Data Availability

The datasets that support the findings of this study are available from the first author upon reasonable request.

## Supplementary Materials

The following supporting information can be downloaded at: https://www.mdpi.com/article/10.3390/pharmaceutics16030403/s1, Table S1: Body weight changes during the 6 weeks period; Figure S1: Representative chromatogram of plasma MDA level analysis of rats; Figure S2: Representative chromatogram of brain MDA level analysis of rats; Figure S3: Representative chromatogram of plasma GSH analysis of rats; Figure S4: Representative chromatogram of brain GSH analysis of rats.
