# Supplementary material for: Drug Repurposing of Metformin for the Treatment of Haloperidol-Related Behavior Disorders and Oxidative Stress: A Preliminary Study
Source: Pharmaceutics. 2024 Mar 15;16(3):403. doi: 10.3390/pharmaceutics16030403 (PMC10974093; doi:10.3390/pharmaceutics16030403)
Supplement: Supplementary file 1 [file pharmaceutics-16-00403-s001.zip › pharmaceutics-2902271-supplementary.pdf]

Table S1. Body weight changes during the 6 weeks period

Group		Week 1	Week 2	Week 3	Week 4	Week 5	Week 6
CTR	1	445	438	444	444	442	442
	2	439	437	445	446	447	447
	3	442	434	440	442	449	449
	4	435	440	440	445	448	448
	5	440	436	438	441	442	442
	6	443	439	440	444	446	446
	7	440	438	438	442	449	449
	8	441	435	439	440	445	445
	9	440	438	437	440	447	447
	10	442	435	437	442	446	446
METF	1	449	446	443	445	445	446
	2	441	437	437	435	436	436
	3	446	441	440	442	443	442
	4	453	447	446	446	448	450
	5	449	445	444	442	445	445
	6	447	443	440	441	443	445
	7	450	447	443	443	445	446
	8	435	432	430	430	435	434
	9	445	442	443	445	444	442
	10	444	439	440	442	440	441
HAL	1	452	448	448	446	445	448
	2	447	445	448	450	448	450
	3	443	440	443	445	445	446
	4	438	435	435	437	440	439
	5	448	443	444	446	446	446
	6	448	446	448	449	448	451
	7	440	439	437	439	441	442
	8	446	447	445	447	448	448
	9	452	448	449	451	449	451
	10	442	444	442	441	445	446
HALMETF	1	449	446	445	447	448	447
	2	438	441	440	443	442	442
	3	451	447	449	448	447	449
	4	436	433	435	438	438	439
	5	446	447	448	446	449	450
	6	440	442	441	443	442	443
	7	445	443	445	448	448	447
	8	447	444	445	447	448	448
	9	453	452	450	449	448	450
	10	443	439	440	440	439	441

CTR – animals receiving distilled water
METF - animals receiving metformin
HAL - animals receiving haloperidol
HALMETF - animals receiving haloperidol and metformin

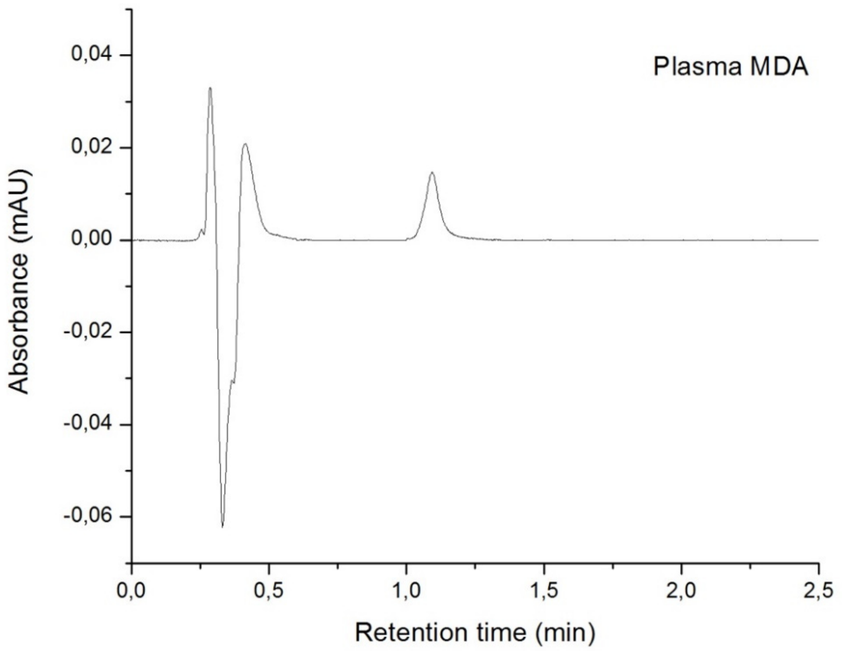

Figure S1. Representative chromatogram of plasma MDA level analysis of rats

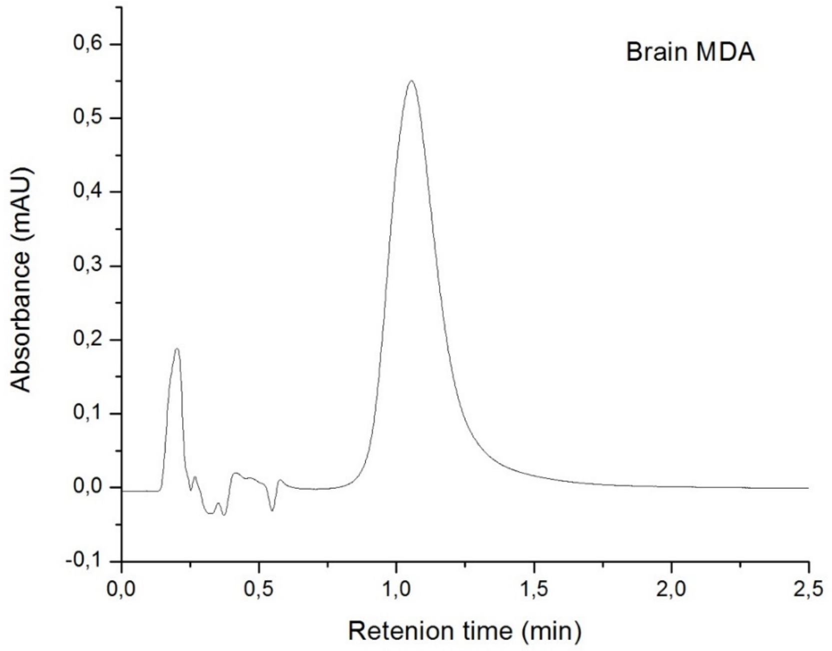

Figure S2. Representative chromatogram of brain MDA level analysis of rats

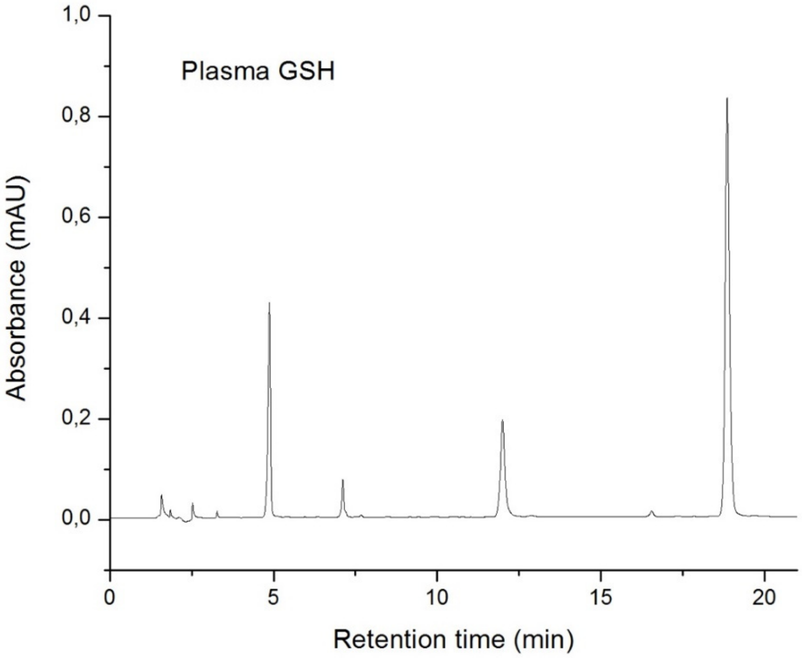

Figure S3. Representative chromatogram of plasma GSH analysis of rats

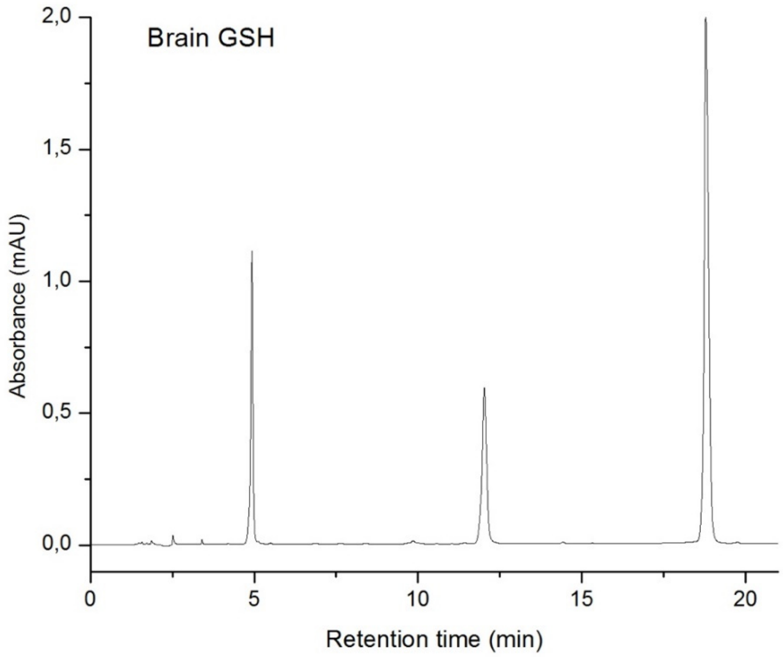

Figure S4. Representative chromatogram of brain GSH analysis of rats
